# Determining the Factors Contributing to Quality of Life of Patients at the Last Stage of Life: A Qualitative Study

**DOI:** 10.5812/ircmj.13594

**Published:** 2013-12-05

**Authors:** Fatemeh Estebsari, Mohammad Hossein Taghdisi, Davood Mostafaei, Ensiyeh Jamshidi, Marzieh Latifi

**Affiliations:** 1 Deparment of Health Education Promotion, School of Public Health, Tehran University of Medical Sciences. Tehran, IR Iran; 2 Department of Health Service Management, School of Health Management and Information, Tehran University of Medical Sciences. Tehran, IR Iran; 3 Community Based Participatory Research Center, Tehran University of Medical Sciences, Tehran, IR Iran

**Keywords:** Quality of life, Self Concept, Patients

## Abstract

**Background:**

Quality- of- life of patients at their last stage of their life are different from that of other people.

**Objectives:**

The aim of this study was to determine the factors contributing to the quality- of- life of patients at their last stage of their life and provide good cares for these patients.

**Patients and Methods:**

This qualitative study was performed by the thematic- framework method of analysis. Twenty three participants including patients, their families, nurses, physicians, psychologists and clergymen were selected sampling. Data were collected by semi – structured interview. We used the thematic framework method to analyze qualitative data.

**Results:**

Seven factors which needed to be considered in the patients’ at last stage quality of life included stress reduction, participation, homecare, education, independency, support, recourses and facilities. According to the findings, the number of these factors may be more than what was mentioned above.

**Conclusions:**

Paying attention to the quality of life at the last stage can be helpful for patients and their families and the special care can be taken for them.

## 1. Background

Addressing the issue of quality of life (QOL) and promoting healthcare have always been a significant matter for health service providers (1). According to the WHO definition, “Health is a state of complete physical, mental, and social well-being not merely the absence of disease” (2). QOL is influenced by demographic, social, economic, cultural, ill- health-related variables (2, 3). Experts believe that the concept of QOL of patients at the last- stage of life is different from that of other people, and is more affected by experiences and activities overshadowed by a life-threatening factor, i.e. Death (3-5). The last-stage of life is a phase during which a person gets close to death (6, 7). Quality of healthcare at the laststage of life presents a major challenge for patients and their families as well as health professionals and policy-makers. Quality of healthcare should be provided equally to all (3, 8-11).

## 2. Objectives

So far in Iran, there has not been a comprehensive study on the last stage QOL of patients. Most studies have dealt with the last stage QOL of patients with chronic diseases or healthy people, and have been primarily concerned with determining the relationship between QOL and factors such as nutrition, physical activity, pain control and the like. This study explored the perceptions and views of participants about the factors contributing to QOL with the aim of providing special care for the patients.

## 3. Patients and Methods

This research is a qualitative study performed in Iran on May 2012-2013. Participants (N = 23) included patients hospitalized at the oncology ward (n = 7), their companions (n = 4), nurses (n = 3), physicians (n = 3) psychologists (n = 3) and clergymen (n = 3) in two major hospitals, namely Taleghani and Shohada-e Tajrish in Tehran. The characteristics of participants are shown in Table 1. Patients with cancer hospitalized at the oncology ward and their close relatives (father, mother, sibling, and offspring) who were actively involved in the care process were selected for the interviews. 

The informed nurses, physicians and psychologists who had at least three or more experience in the oncology ward were invited to interviews. Clergymen who had previously collaborated with hospital were also invited. In addition to speaking in Farsi, willingness to participate in the study and tendency to answer the questions were the main criteria. The two hospitals were selected because of referring the patients with various diseases and from diverse cultures and authority. Interviewers were members of the research team including a nurse (female) and a physician (male).They have working with cancer patients in hospital for more than three years and were highly skilled in doing interviews. 

 The sampling method was homogeneous in the process of qualitative research (12, 13) and the sample size was based on the data saturation. Therefore, the selection of eligible cases continued until all of data were collected (Data saturation phenomenon) (14, 15). A total of 25 people were interviewed. Participants who refused to answer the questions during the interview were excluded from the study (Two people). Twenty three interviews were conducted. The method of collecting data was semi-structured interviews, beginning with a number of general questions about the topic of the study. Consulting advisors and academic experts, the researcher designed the questions based on the related studies and texts. Concerning the objectives of the study, some of the questions were previously designed. The questions were related to the factors contributing to QOL of patients at the last-stage of their life. Efforts were made to win the confidence of participants in the study by explaining the objectives of the study. They were ensured that all information was completely confidential and collected only for research purposes. The interview took from 30 to 45 minutes. Participants agreed that the conversations be recorded, except one . Every participant was interviewed only once. The interviews with the patients were conducted privately. Interviews with their accompanying relatives were conducted in a place designated by them (the hospital courtyard, hall, and hospital’s restaurant). Appointments with the physicians, nurses, psychologists, and clergymen were arranged in advance at their preferred location (their resting room, treatment, conference rooms, pavilions, or office). The data analysis was done in five steps, namely "familiarizing, identifying a thematic framework, indexing, charting, mapping, and interpreting" (16-18). At familiarizing stage, in order to be familiar with the content of data and note key ideas and recurrent theme, the researcher immersed into the data and read the transcripts thoroughly. All interviews were transcribed in fewer than 24 hours. The researcher got familiar with the range and diversity of the content and issues. At identifying stage, to produce a detailed index of the data, the researcher divided the textual data into understandable chunks (key issues, concepts, and themes). This was done for subsequent retrieval and exploration and examining the data. Then, the codes were fully defined and borders specified. At indexing stage, after searching and identifying themes, codes with similar meanings were collected and core concepts were formed. According to this framework, all the transcriptions were reviewed, annotated, and categorized. Concepts, contradictions, theories, experiments, and research were compared, and the patterns and relationships were inferred from the findings. Simultaneously, data were encoded. Then, the key concepts and themes were identified based on the thematic framework method. At charting stage, the charting process involving a considerable amount of abstraction and synthesis was done. A collection of some concepts were presented as main ones. The main concepts were assigned to the primary codes. Finally, at mapping and interpreting stages, initial framework including 14 concepts was developed . These concepts were reduced to seven ones in final analysis.

The analysis can be done via Atlas.ti or otehr popular software programs such as Microsoft Word (18). The latter was used in the current study. This study has been reviewed and approved by the ethics committee of TUMS and the ethical code number is 12638. The study tried to ensure the participants. For this purpose, interviewers introduced themselves to participants, the research objectives were clearly stated and the participants were assured that the information will remain confidential. Thus, the researchers did not identify participants by name in any research output, using just the information obtained from interviews. In case of lack of collaboration and withdrawal from the study, there wasn’t any potential harm to the participants. Researchers made necessary arrangements with hospitals and nursing units. In interviews with patients and their relatives, no sensitive terms like last-stage or similar words were used.

**Table1.** Characteristics of Participants (N = 23).

Work Experience, y	Education, No.			Age, y	Gender		Characteristics of Sample
	Higher	Secondary	Primary		Female	Male	
**--** ^**a**^	3	2	2	27 - 64	4	3	Patients
**--**	2	2	--	16 - 48	2	2	Patients Companions
**3 - 10**	3	--	--	31 - 41	2	1	Nurse
**3 - 18**	3	--	--	44 - 57	--	3	Physician
**3 - 15**	3	--	--	39 - 41	2	1	Psychologist
**6 - 15**	3	-	-	47 - 51	-	3	Clergymen

^a^--; Not available

## 4. Results

The factors contributing to the last stage QOL of patients included seven main concepts, and 28 themes were recognized (Table 2). 

### 4.1. Reducing Stress

The eight themes were identified as factors which led to relieving patients’ stress.

#### 4.1.1. Not Leaving the Patient Alone

Participants believed that not leaving a patient alone, spending time with their spouse, children, relatives, acquaintances, friends, and colleagues could significantly alleviate stress and ultimately improve patients' state.

#### 4.1.2. Providing a Stress-Free Environment

Patients at the last-stage need rest more than any other things. A calm and cheerful environment impacts on boosting morale.

#### 4.1.3. Avoiding Talking about the Unfavorable Events

In order for the patient to enjoy life, we’ll do whatever we can to avoid stress and anxiety, for example, never talking about treatment expenditures, cost of living, or the cost of children’s education.

#### 4.1.4. Concealing the Truth

Patients’ companions expressed that they preferred not to mention the true diagnosis or details of the disease to their patient to reduce stress and anxiety.

#### 4.1.5. Resorting to Religious Beliefs, Praying, and Asking for Forgiveness

Another factor that contributed to reduce the patient's stress and make them cope with their illness was relying on religious beliefs and attitude to be recovered from the disease. Seeking forgiveness from friends and family before passing away was another technique practiced by the patients in the study.

#### 4.1.6. Resorting to the Clergy

In this study, the major preoccupation of participants was questions about death, the other world, and the life after death. This state appears more predominant in case of sickness and hospitalization.

#### 4.1.7. Seeking Counseling and Psychiatric Services

The psychologists believed that coming to terms with incurable diseases, especially cancer, is difficult for patients and their families. These patients and their families undergo severe pressure and stress during the treatment and even after it.

#### 4.1.8. Planning for after Death

Participants in the study believed that people in every situation, whether healthy or sick, consider death and try to prepare for it.

### 4.2. Participation and Assistance

The following themes were identified including: participation of the patients at their last-stage of life and their family. Patients will feel calm when their treatment is shared. This will also inhibit the vagueness and hopelessness. Family participation in the treatment process was considered as substantially effective factor for the patients’ QOL.

### 4.3. Home Caring

The themes included

#### 4.3.1. Resuming Treatment in the Familiar Environment of Home and Family

In this study, most participants stated that home treatment is tremendously effective in increasing the QOL of patients.

#### 4.3.2. Relieving Depression

The physicians believed that resuming the treatment process at home, rather than in the isolated and impersonal environment of the hospital, can alleviate depression. When at home, the patient is once again placed in a familiar environment where they see their beloved ones. Psychologists declare that the hospital environment causes stress, and most of the patients want to escape from this situation without completing the course of their treatment.

#### 4.3.3. Reducing Costs

One major concern for patients and their families, according to the physicians and nurses, is the hospitalization expenditures. Providing care services by trained nurses at home causes a considerable amount of budget be saved for the patient and their family.

### 4.4. Training

The following topics include:

#### 4.4.1. Training patients

Participants ,in the study, stated that self-care training is one of the factors that can improve QOL.

#### 4.4.2. Training Families

The majority of patients and families need to learn homecare techniques and skills.

#### 4.4.3. Training the Personnel

Another main concern raised by patients, families, and medical staff was the need for trained nurses.

#### 4.4.4. Raising the Society’s Awareness

Patients’ companions claimed that communities should be aware of behaviors such as patronization and compassion to patients with cancer and promote them.

### 4.5. Independence

It refers to the issue of self-reliance and having control over the life, environment and events. Being independent in doing personal tasks was a key concerns raised by the patients participating in the study. Moreover, having control over their life, and the events in their environment impacts greatly on their QOL.

### 4.6. Support

The following themes were identified: 

#### 4.6.1. Support from Family and Friends

 Participants of the study deemed support from family and friends important for the QOL of patients. They believed that family and friends are the most crucial sources of emotional and psychological support. In addition, participants called for the government to provide financial support for the cost of drugs. Financial support is necessary for patients and families. Some participants suggested that private and non-private organizations support financially.

### 4.7. Facilities and Resources

The five themes included increasing the number of non-private hospitals, equipping them with specialized and diagnostic machines, increasing the staff especially experienced and trained nurses, providing chemotherapy drugs, and proper insurance coverage. Patients and their families were not satisfied enough with the equipment of hospitals such as specialized and diagnostic devices and machines. Supply of chemotherapy drugs, regardless of their cost, is one of the basic problems raised by patients and their companions. Another factor affecting the QOL of patients was quality of insurance services. In general, the performance of insurance organizations was unacceptable from the participants’ point of view.

**Table 2.** The Factors Contributing to QOL of Patients Emerged as seven main Concepts

	Key Concepts	Themes
**1**	Reducing stress	(a) not leaving the patient alone, (b) providing a free-of-stress environment, (c) avoiding upsetting events, (d) concealing disquieting facts, (e) resorting to religious beliefs, praying, and asking for forgiveness, (f) asking questions of a cleric, (g) seeking counseling, (h) planning for after death.
**2**	Participation and assistance	(a) participation of patient in their last stage in their treatment, (b) participation of their family in patient’s treatment
**3**	Home care	(a) Resuming treatment in the familiar environment of home and family, (b) Relieving depression, (c)Reducing costs
**4**	Training	(a) Training patients, (b) Training families, (c) Training the personnel, (d) Raising the society’s awareness
**5**	Independence	(a) self-reliance, (b)having control over their life, and the environment and events.
**6**	Support	(a) Support from family and friends, (b) financial support from the government to provide the costs of drug and cost of living, (c) Support from private and non-private organizations or community groups
**7**	Resources and facilities	(a) Increasing the number of public hospitals, (b) equipping public hospital with specialized and diagnostic machines, (c)increasing the staff especially experienced and trained nurses, (d) providing chemotherapy drugs, (e) proper insurance coverage.

## 5. Discussion

According to the interviewees, several factors shape the QOL of patients at their the last-stage. Reducing stress and anxiety is one of the factors for providing better QOL for patients at the last-stage of life. Creating a stress-free environment, not mentioning the disturbing events and hiding distressing realities, and not leaving the patient alone were some of the issues identified in this study. A study by Mak and Clinton (6) identified factors such as spending time with spouse, children, friends, and family is effective for enhancing the QOL of patients facing death. Findings of the present study are consistent with these matters. Being faithful, saying prayer, and asking for forgiveness and the presence of a cleric were some other factors to provide optimum QOL for patients at the last-stage. Seeking forgiveness, saying prayer, and talking with a cleric before death were some of the needs of the participants, which should to be respected. Patrick et al. (5) confirmed that appreciating the patients’ religious convictions and their demands and expectations before death are some of the factors that makes them calm (5, 19). Referring to the psychologists and therapists is not common and they are referred to only if the physician recommends. However, all patients, particularly those have depression, and their families need counseling. Counseling must begin as soon as the disease is diagnosed. These patients need to be informed about efficacy of the treatment to boost their morale. Every human has the right to have good life and they must be supported to enjoy (20, 21). Preparing for death by planning in advance, has been recognized as one of the elements that leads to an enhanced QOL of patients. Preparing for death is advisable for these patients and their families as it can anticipate actions that are based on sentiment rather than reason (4). Still, another factor that is effective for the QOL of patients was relatives’ cooperation and patients’ participation. Letting the patient do some of his personal tasks can give them a sense of accomplishment and usefulness. Similar studies have also come up with the conclusion that mutual interaction the patients and their family can be more effective on QOL (3, 22). The cost of hospitalization services for patients with cancer is increasingly exorbitant. Therefore, if these services provide at home either by a family member or a nurse, it is strongly recommended that the patients be transited to home. This issue causes the costs reduce and makes the patients and their family calm and tranquil. In order for these patients to have appropriate treatment at home, first, their psychological, social, spiritual, and economic needs must be perceived. Secondly, the patient and their family need to be trained properly so that the home-based treatment will be as efficient as possible. Designing an educational program and setting out homecare guidelines are the first steps for the transmission of treatment from hospital to home (23). According to the participants, training the patients for self-care and family members for assisting the patient are the initiatives thought to be practical and worthwhile (23). Besides, physicians and the nursing staff have to to be trained and receive adequate information about last-stage patients. Last-stage patients are different from others because they are more sensitive and need gentle handling. Factors such as psychological support, counseling, professional function toward patients and their families, respect and kindness, common understanding, teamwork, and medical support were are effective on the QOL of patients at the last-stage. Budis et al. (3) and Levin et al. (24) emphasized on the interdisciplinary nature of care and explained that the training program for taking care of patients at the last stage of life should include the concepts of good health care, last-stage detection, detecting sorrow and melancholy, advanced care planning, pain management, emotional and spiritual care, and taking care of the care-takers (3, 24, 25). Society should change their views toward patients with cancer. In this regard, the role of the mass media, particularly television and radio, were considered more notable. Independence is considered as a factor that could impact on the QOL of last-stage patients. Patrick (5) and Gallagher (22) found that the participants in their study wanted to have control over their lives and their environment (5, 11, 22). The strategies for gaining control over life included getting support from professional staff, identifying individuals’ coping skills, ensuring patient and family participation in care-taking, considering the family as a unit, and having open communication. Encouraging patients to participate in self-care program is a way of empowering and increasing QOL (25, 26). Supporting was also recognized as a factor that could elevate the QOL of patients. Supporting may come from political, economic, social, cultural and environmental organizations and may include raising awareness, training, assisting mutually, counseling and intervening. Thacker et al. (26) discovered that recruiting the nurses to take care of patients at the last-stage of life could increase the QOL (24, 26). The most important kind of support include psychological one by family and friends; governmental support includes financing healthcare costs; support from social and charity organizations such as private, non-private, insurance companies and NGOs. Economic problems and medicine supply were the major concerns of patients and their families. Governmental support, negotiation with insurance companies, revising the insurance laws particularly special patients’ insurance can be beneficial. Some of the care services that must be provided for patients at the last-stage life by health care system based on Joint Commission International and Accreditation Standards (COP. 7) include sympathetic and feeling treatment, respecting the religious values and cultural preferences of patients, having patients and their families participate in all possible aspects of care-taking, and responding to their mental, emotional, and spiritual concerns (27). Factors obtained from this study are consistent with hospital accreditation standards for patients. Everyone wants to die comfortably. Therefore, patients at the last-stage of life deserve the same attention as a healthy individual does, and all aspects of their life and their relatives and family must be intended to. It is hoped that the information presented in this paper be helpful to healthcare policy-makers and planners for providing a better QOL for patients at the last-stage of life. Need to mention that this study was qualitative and conducted on a small scale with a small sample size, lead to reach the limited findings. Different perspectives of clergymen and psychologists that play an important role in health promotion and quality of life of patients at their last stage provides valuable information that can be considered as an advantage of the study. Other strong point of the study was to interview with different groups of people including patients at the last -stage of their life. Interviewing with the patients at the last -stage of life and their companions is difficult which requires specific communication skills.
